# Post-Transplant Tremor: Characteristics and Differences Based on Sex and Post-Transplant Therapy

**DOI:** 10.3390/neurolint18030056

**Published:** 2026-03-17

**Authors:** Srdjana Telarovic, Maja Vrdoljak Pazur, Nikolina Zupancic, Anamarija Strajduhar, Irma Telarovic

**Affiliations:** 1 School of Medicine, University of Zagreb, Salata 3b, 10000 Zagreb, Croatia; 2 Department of Neurology, University Hospital Centre Zagreb, Kispaticeva 12, 10000 Zagreb, Croatia; 3 University Hospital for Infectious Diseases “Dr. Fran Mihaljevic”, Mirogojska 8, 10000 Zagreb, Croatia; 4 Department of Anesthesiology and Intensive Care, University Hospital Merkur, Zajčeva 19, 10000 Zagreb, Croatia; 5 Clinic for Pulmonary Diseases, University Hospital Centre Zagreb, Jordanovac 104, 10000 Zagreb, Croatia; 6 Department of Radiation Oncology, University Hospital Zurich, University of Zurich, Raemistrasse 100, 8091 Zurich, Switzerland

**Keywords:** post-transplant tremor, calcineurin inhibitor-induced tremor, tremor in females

## Abstract

**Background/Objectives**: Kidney transplantation is the standard of care for the majority of patients with end-stage kidney disease. Neurological complications are common, and among them, tremor is very frequent and usually attributed to immunosuppressive drug toxicity. **Methods**: In this retrospective study, we investigate the incidence and characteristics of tremor in kidney transplant patients and analyze its occurrence with respect to a multitude of demographic and clinical parameters, thereby aiming to confirm the role of calcineurin inhibitor-induced neurotoxicity and to identify other putative predictive factors. Furthermore, we characterize post-transplant tremor with the goal of identifying its clinical features and determining the impact on quality of life. **Results**: A total of 129 kidney transplant recipients were screened; six patients were excluded due to a history of movement disorders prior to kidney transplantation. In total, 123 patients were included in the final analysis—69 male (56%) and 54 female patients (44%), with a median age of 50. A total of 36% (46 patients) developed tremor in the post-transplant period. Using both univariable and multivariable analyses, we found that female sex and tacrolimus use were independently associated with the development of post-transplant tremor. In addition, multivariable analysis identified an association between younger age and post-transplant tremor. Furthermore, we observed a trend in the duration of symptoms in relation to the calcineurin inhibitor choice. **Conclusions**: Despite a relatively high prevalence (36%), post-transplant tremor does not significantly impact the QoL and spontaneously resolves within 1 year in adult kidney transplant recipients. Female sex and tacrolimus were identified as independent predictors of post-transplant tremor in renal transplant recipients.

## 1. Introduction

Kidney transplantation is the standard of care for the majority of patients with end-stage kidney disease, with superior outcomes in terms of quality of life (QoL) and survival in comparison to dialysis [1,2]. Both graft and patient survival have been steadily improving in recent decades, with a current 5-year patient survival rate of around 80% for deceased donor kidney recipients and over 90% for living donor kidney recipients [2]. However, due to the lifelong necessity for immunosuppressive therapy and often present comorbidities, transplant recipients remain a population at risk for serious complications.

Neurologic complications are common after solid organ transplantation and are a significant cause of mortality and morbidity, affecting between 10 and 85% of patients [3,4,5]. Out of those, renal transplant recipients are among the most affected, with between 8 and 30% experiencing neurologic problems. Advanced atherosclerosis-induced cerebrovascular events are the most common; however, a wide range of complications may occur—ranging from headaches and disturbances of consciousness and behavior to movement disorders, convulsions and encephalopathies [3,6,7].

Tremor is a frequent neurologic complication of solid organ transplantation and is usually attributed to immunosuppressive drug toxicity. Among the different classes of immunosuppressant drugs, calcineurin inhibitors (CNIs)—tacrolimus and cyclosporine—are most often implicated in neurotoxicity. In patients experiencing CNI-induced neurotoxicity, tremor is the most common neurological finding [8,9].

Although both CNIs have a similar neurotoxic range, tremor is significantly more common in patients receiving tacrolimus compared to cyclosporine, with occurrence rates varying between 40 and 54% for patients on tacrolimus, compared to 20–34% for patients on cyclosporine [8,9,10]. Notably, the majority of neurologic complications associated with immunosuppressive drugs occur within one month after surgery, which can partially be attributed to high doses of immunosuppressive agents typically administered in this timeframe. At the same time, several studies have shown that the occurrence of CNI-induced neurotoxicity does not follow a simple dose–response relationship, thereby indicating that other factors might play an important role [5,8,9].

The severity of post-transplant tremor varies; some patients experience mild postural tremor and report no impact on the QoL, while others suffer from severe, debilitating tremor which prompts an immunosuppressive regimen modification [10,11]. In spite of the growing recognition of its importance, there is currently a paucity of comprehensive studies focused on identifying the characteristics and potential predictors of post-transplant tremor and its impact on QoL.

In this retrospective study, we investigate the incidence and characteristics of tremor in kidney transplant patients. We analyze the occurrence of tremor with respect to a multitude of demographic and clinical parameters, thereby aiming to confirm the causal role of CNIs in the occurrence of post-transplant neurological complications and to identify other putative predictive factors. Furthermore, we characterize post-transplant tremor with the goal of identifying its clinical features and determining the impact on QoL in our patient population.

## 2. Materials and Methods

### 2.1. Patient Selection

We screened all patients who underwent kidney transplantation at the Division of Urology and the Division of Nephrology, Hypertension and Dialysis of the University Hospital Centre Zagreb, Croatia, between 2003 and 2013. Patients with a history of movement disorders prior to kidney transplantation were excluded from the study. The study was conducted in accordance with the Declaration of Helsinki and approved by the Ethics Committee of the Clinical Hospital Centre Zagreb. Informed consent was obtained for all patients prior to inclusion.

### 2.2. Patient Assessment

Demographic and clinical characteristics of patients (age at transplantation, sex, underlying kidney disease, hypertension, duration of dialysis, donor type, immunosuppressive pharmacotherapy, and tremor appearance and characteristics) were collected from medical records. In accordance with the institutional guidelines developed by the multidisciplinary team for kidney transplantation, all kidney transplant recipients are systematically screened for the presence of neurological side effects, including tremors. Routine assessments occur every three months for asymptomatic patients, with more frequent evaluations conducted as clinically indicated.

At the timepoint of data collection, all patients with a documented history of post-transplant tremor additionally underwent a neurological evaluation conducted by physicians-in-training (M.V.P., N.Z., and A.S.) under the supervision of a movement disorder expert (S.T.). The evaluation included targeted patient history (familial history of tremor, history of movement disorders prior to kidney transplantation, age at onset, time at onset relative to kidney transplantation, and duration of tremor) and if applicable, tremor assessment based on the recommendation by the Tremor Task Force of the International Parkinson and Movement Disorder Society [12]. The categories assessed were: tremor type (action, rest, and action and rest), tremor distribution (focal: only one body region is affected; segmental: two or more contiguous body parts in the upper or lower body are affected; hemitremor: one side of the body affected, generalized) and activities of daily living (level of impairment: no impairment, mild, moderate, or severe).

### 2.3. Statistical Analysis

Statistical analysis was performed using GraphPad Prism, version 8 (GraphPad Software Inc., San Diego, CA, USA) and R version 3.6.2 (R Foundation for Statistical Computing, Vienna, Austria). Significance was defined as a *p* value < 0.05. To reduce the risk of a type II error, we used a standard, non-corrected significance level of 0.05 throughout the study given its explorative nature. Continuous variables are represented as median (range). Categorical variables are represented as a count (percentage). Variables with missing values are marked by an asterisk and analyzed using available-case analysis. Data distribution was tested for normality with the Shapiro–Wilk test. Continuous variables were then analyzed using the t-test (for data following a normal distribution) or the Mann–Whitney test (for data not following a normal distribution). Categorical variables were analyzed using the Chi-square test (for 2 × 2 contingency tables with cell numbers ≥ 5), Fisher’s exact test (for 2 × 2 contingency tables with cell numbers < 5) or the Freeman–Halton extension of Fisher’s exact test (for contingency tables > 2 × 2). To identify potential predictors of post-transplant tremor occurrence, we performed multiple logistic regression, with the presence or absence of tremor after transplantation as the dependent variable. All variables with a *p* value < 0.2 resulting from the comparison between patients with and without post-transplant tremor (age at transplantation, sex, duration of dialysis, and CNI) were entered into the multiple logistic regression model. The variance inflation factor (VIF) was used to exclude multicollinearity. The Hosmer–Lemeshow test was used to evaluate the goodness of fit.

## 3. Results

### 3.1. Demographic and Clinical Characteristics of Patients

A total of 129 kidney transplant recipients were included in the screening. Six patients were excluded from further analysis due to a history of movement disorders prior to the kidney transplantation. Demographic and clinical characteristics of the remaining 123 patients who were included into the final analysis are summarized in Table 1.

Our cohort included 69 male (56%) and 54 female patients (44%), with a median age of 50 (range 14–71) at the time of kidney transplantation. The leading causes of kidney failure were glomerulonephritis and other glomerular diseases (40 patients, 33%), followed by polycystic kidney disease and other tubulointerstitial diseases (24 patients, 20%), hypertensive or diabetic nephropathy (18 patients, 15%) and congenital anomalies (18 patients, 15%). In 20 patients (16%), kidney failure was of unknown etiology. The majority of patients reported a history of hypertension (95 patients, 80%) and dialysis (120 patients, 99%), with a median duration of 47 months (range 3–212). Furthermore, the majority of patients received a transplant from a deceased donor (118 patients, 96%). Immunosuppressive triple therapy according to the national guidelines [13] and in accordance with the 2009 Kidney Disease: Improving Global Outcomes (KDIGO) clinical practice guidelines for kidney transplantation [14] included: a glucocorticoid (prednisone, received by all patients), an antimetabolite (mycophenolate mofetil, received by 109 patients, 95%) and a CNI (cyclosporine or tacrolimus, received by all patients; 54% of patients received cyclosporine and 46% received tacrolimus). A total of 10% of patients also received everolimus as part of their treatment, primarily in cases where CNI therapy had to be stopped due to high-grade toxicity.

### 3.2. Predictors of Tremor

Comparisons between kidney transplant recipients revealed differences between the patients who developed tremor after transplantation (group T: 46 patients, 37%) and those who did not (group NT: 77 patients, 63%) (Supplementary Figure S1). In the T group, the majority of patients (57%) were female, compared to a minority (36%) in the NT group (*p* = 0.029) (Supplementary Figure S1A). No significant differences between the two groups were detected with respect to age at transplant, the underlying kidney disease, history of hypertension or the donor type. Prior to kidney transplantation, patients in the NT group spent more time on dialysis compared to the patients in the T group (54 months, range 3–169, in the NT group vs 36 months, range 7–212, in the T group; *p* = 0.017). With regard to the post-transplant immunosuppressive therapy, inclusion of everolimus, glucocorticoids and antimetabolites did not significantly differ between the groups. The choice of the CNI, however, did reveal a significant difference in the T group compared to the NT group: patients in the T group were more likely to have received tacrolimus (74% tacrolimus; 26% cyclosporine) while the majority of patients in the NT group received cyclosporine (29% tacrolimus; 71% cyclosporine) (*p* < 0.001) (Supplementary Figure S1B).

To assess these variables as putative independent predictors of the occurrence of tremor after kidney transplantation, we performed multiple logistic regression analysis (Supplementary Table S1), which indicated that younger age at transplantation (*p* = 0.041, odds ratio (OR) = 0.961, confidence interval (CI) = 0.924–0.998, and variance inflation factor (VIF) = 1.091), female sex (*p* = 0.009, OR = 2.607, CI = 1.424–10.190, and VIF = 1.047) and the use of tacrolimus (*p* < 0.001, OR = 3.782, CI = 2.554–18.130, and VIF = 1.102) independently predicted tremor occurrence in our cohort. The *p* value of the Hosmer–Lemeshow test was 0.315, indicating an acceptable goodness of fit.

### 3.3. Characteristics of Tremor

**Figure 1:**
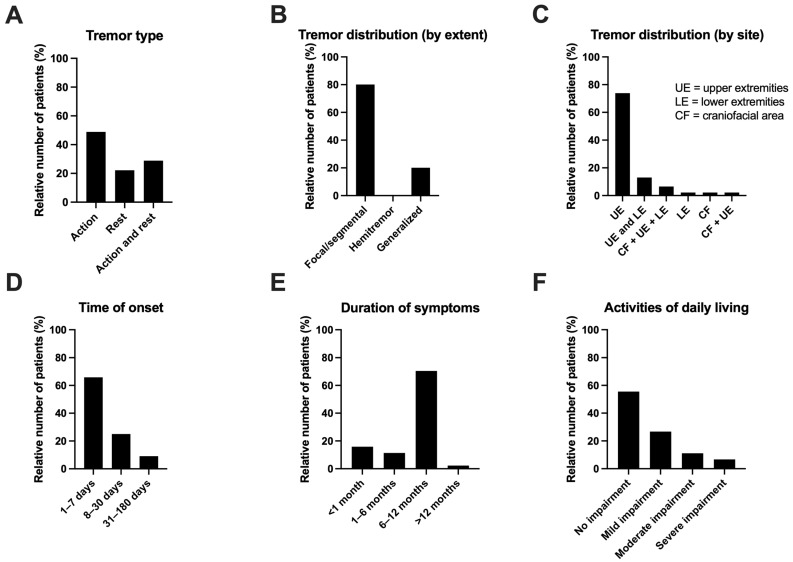
Clinical characteristics of post-transplant tremor. ((**A**): Tremor type; (**B**): Tremor distribution (by extent); (**C**): Tremor distribution (by site); (**D**): Time of onset; (**E**): Duration of symptoms; (**F**): Activities of daily living.).

A total of 46 patients (37%) developed tremor after transplantation in our study. The clinical characteristics of tremor are summarized in Figure 1 and Table 2. The majority of patients experienced action tremor (49%) followed by a combination of action and rest tremor (29%) and an isolated tremor at rest (22%) (Figure 1A). Considering the distribution by extent, tremor was primarily focal or segmental (involving one or more contiguous body parts in the upper or lower body, detected in 80% of patients), followed by generalized tremor (20%) (Figure 1B). No patients experienced hemitremor. Considering the distribution by site, the majority of patients had isolated upper extremity involvement (74%), followed by the involvement of both upper and lower extremities (13%) and a combination of upper extremities, lower extremities and the craniofacial area (7%) (Figure 1C). With respect to the time of onset, 66% of patients reported the occurrence of tremor within 1 week after transplantation. A further 25% experienced symptoms within 1 month, while the remaining 9% developed symptoms between 1 and 6 months after transplantation (Figure 1D). Symptoms resolved within 6 to 12 months for the majority of patients (71%) (Figure 1E). Regarding the activities of daily living, “No impairment” was the most common assessment (55%), followed by mild, moderate and severe impairment (27%, 11% and 7%, respectively) (Figure 1F).

**Figure 2:**
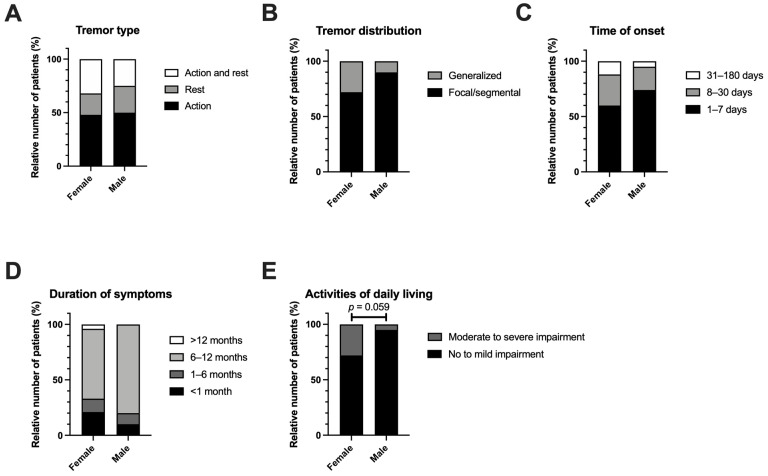
Characteristics of tremor with respect to sex. ((**A**): Tremor type; (**B**): Tremor distribution; (**C**): Time of onset: (**D**): Duration of symptoms; (**E**): Activities of daily living).

Based on the observation that female sex might contribute to the occurrence of tremor after transplantation, we next investigated potential differences between men and women with regard to tremor characteristics (Table 2 and Figure 2). No significant differences were detected. However, we observed a trend towards a higher degree of impairment among women (seven female patients reported “Moderate impairment” or “Severe impairment” vs one man; *p* = 0.059) (Figure 2E).

Furthermore, we compared tremor characteristics with respect to the choice of CNI (Supplementary Table S2) and age at transplant (Supplementary Figure S2). Similarly, we detected no significant differences. However, intriguingly, none of the patients who received cyclosporine reported symptom resolution within the first 6 months after transplantation. In contrast, 20% of the patients treated with tacrolimus experienced tremor cessation within the first month after transplantation, while an additional 17% reported symptom resolution occurring between 1 and 6 months after surgery. With respect to the age at transplant, younger age was associated with the combined (action and rest) tremor type (median age for rest, action and combined tremor was 46, 52, and 37, respectively) (Supplementary Figure S2A).

## 4. Discussion

Post-transplant tremor is a commonly reported yet insufficiently characterized neurologic complication after solid organ transplantation. Immunosuppressant drugs, in particular CNIs (i.e., tacrolimus and cyclosporin), seem to play the major causal role. Post-transplant tremor is significantly more common in patients receiving tacrolimus compared to cyclosporine, with occurrence rates varying between 40 and 54% for patients on tacrolimus, compared to 20–34% for patients on cyclosporine [8,9,10]. However, heterogeneity across studies regarding tremor onset, severity, and duration, combined with the absence of a clear dose–response relationship, suggests a rather multifactorial etiology, with other putative causal factors remaining unknown [5,8,9,15,16,17].

In this study, we performed a comprehensive retrospective analysis of tremor in kidney transplant recipients. Our cohort included 129 kidney transplant recipients—69 male (56%) and 54 female patients (44%), with a median age of 50 (range 14–71). Six patients were excluded due to a history of movement disorders prior to kidney transplantation. Out of the remaining 123 patients, 36% (46 patients) developed tremor in the post-transplant period.

Since this is a retrospective study, the possibility of recall bias cannot be excluded. However, in accordance with the institutional guidelines developed by the multidisciplinary team for kidney transplantation, all kidney transplant recipients were systematically screened for the presence of neurological side effects, including tremors, as part of routine follow-up exams. While this systematic screening strategy minimizes recall bias regarding the occurrence of tremor, the specific neurological characteristics of the tremor were not systematically documented at the time of onset but were instead assessed during a targeted neurological evaluation at the point of data collection. This approach may have introduced recall bias, particularly for patients whose symptoms resolved in the distant past.

Univariable and multivariable analyses identified female sex and tacrolimus use as independent risk factors for post-transplant tremor. Multivariable modeling also suggested an association with younger age (*p* = 0.041); however, this finding warrants caution as the age was not significantly different between the T and NT groups in univariable analysis (*p* = 0.088). In line with our data, both Venute et al. and Tornatore et al. reported women receiving tacrolimus after kidney transplantation as having a significantly higher neurologic adverse effect score compared to their male counterparts [16,18,19]. Notably, and in contrast to our investigation, none of the previous studies focused on tremor alone but rather assessed a composited neurologic adverse effect score, defined as a combination of headache, tremor and/or insomnia. Evidence linking age to post-transplant tremor is limited and inconclusive [16]. In contrast to our data, Fernandez Rivera et al. found a significant association between older age and tremor development [20]. This discrepancy may be attributed to differences in study populations; specifically, their cohort consisted exclusively of patients on tacrolimus and featured a higher mean age (57 years) and a greater proportion of male participants (72%) compared to our study.

Our study confirmed the known association of CNI with the occurrence of post-transplant tremor, in particular in patients treated with tacrolimus [8,9,10]. Importantly, an in-depth analysis of pharmacokinetic parameters was out of the scope of this study. Furthermore, the choice of CNI was at the discretion of the attending nephrologist, following an individualized approach that accounted for patient-specific comorbidities, such as diabetes and obesity. The potential impact of these additional variables was not systematically evaluated as part of this study. As such, potential confounders related to drug selection, formulation and concentration cannot be excluded. However, findings from other studies, which primarily focused on these aspects, align with our own [21].

Interestingly, in the univariable analysis, duration of dialysis prior to kidney transplantation was significantly shorter in patients who developed post-transplant tremor compared to those who did not. Although the statistical significance was not maintained in the multivariable analysis, this intriguing finding warrants further investigation; duration of dialysis might be a surrogate marker for other important variables which have not been assessed in our study. For example, prolonged dialysis duration may reflect challenges in identifying a suitable donor, which is potentially linked to specific immunophenotypes. Other variables, such as the presence or absence of specific comorbidities that could also affect the choice of immunosuppressive therapy, should also be considered.

Despite a relatively high prevalence of post-transplant tremor (36%) in our cohort, the majority of affected patients (55%) reported “No impairment” in their activities of daily living, thus indicating that post-transplant tremor does not significantly impact QoL, which aligns with previous studies [17]. However, these findings should be interpreted with caution, as the QoL in our study was assessed indirectly by a questionnaire focused solely on the activities of daily living. To increase the generalizability of our findings, further studies should include extended, validated QoL questionnaires, such as SF-36 [22].

Furthermore, we characterized post-transplant tremor according to its clinical presentation: the majority of patients presented with an action tremor (49%), primarily localized to the upper extremities (74%), manifesting within the first week (66%) and resolving between 6 and 12 months post-transplantation (71%). Interestingly, our study identified a trend regarding symptom duration in relation to the choice of CNI. In the cyclosporine group, post-transplant tremor persisted for a minimum of 6 up to a maximum of 12 months for all patients. In contrast, in the tacrolimus group, post-transplant tremor resolved within 1 month for 20% of the patients and within 6 months for an additional 17%. The significance of this finding, as well as a potential underlying mechanism, should be further investigated. Interestingly, preclinical data suggest that tacrolimus might exhibit neurotrophic and nerve-protective properties, potentially promoting neural regeneration in the long term. In contrast, cyclosporine has been associated with a reduction in axonal regeneration [23].

Some studies identified a predominantly postural tremor in their subjects [17], while others reported a combination of postural and action tremor [15]. In our study, the majority of participants experienced an action tremor isolated to the upper limbs. This presentation makes the post-transplant tremor clinically similar to essential tremor (ET), the most common tremor type overall. However, post-transplant tremor is distinguished from ET by its clear association with the initiation of immunosuppressive therapy and its spontaneous regression in most cases after 6–12 months, as confirmed by our findings. Furthermore, ET frequently involves a hereditary component, which is normally absent in the case of the post-transplant tremor.

When tremor appears as an isolated neurologic adverse effect, our research suggests it points toward a favorable neurological outcome. Conversely, if a patient concurrently presents with other symptoms—such as headaches, insomnia, confusion, blurred vision, photosensitivity, or nightmares—it may indicate the development of encephalopathy, necessitating further diagnostic workup and diligent patient monitoring [16].

Altogether, these findings underscore the importance of early tremor recognition in the setting of kidney transplantation, in particular in younger patients and women. It is essential to monitor for its emergence in the early weeks following transplantation and to reassure patients that, in most instances, this is a transient disorder. However, if there is no improvement over a prolonged period and/or if the tremor significantly impairs QoL, patients should be referred to a specialized Movement Disorders Clinic to rule out comorbid movement disorders and, if necessary, receive symptomatic therapy.

Taken together, the findings of this study have direct implications for improving clinical practice and contribute to a deeper understanding of the incidence of tremor following kidney transplantation, thus providing a foundation for further research into this relatively common, yet still under-investigated, issue.

## Figures and Tables

**Table 1:** Demographic and clinical characteristics of patients with and without post-transplant tremor.

	All (*n* = 123)	Tremor (T) (*n* = 46)	No Tremor (NT) (*n* = 77)	*p* Value
Age (y), median (range)	50 (14–71)	48 (16–70)	52 (14–71)	0.088
Sex, *n* (%)				**0.029**
Male	69 (56)	20 (43)	49 (64)	
Female	54 (44)	26 (57)	28 (36)	
Familial history of tremor, *n* (%)	[*n* = 46] *		No data	NA
Yes	0 (0)	0 (0)		
No	46 (100)	46 (100)		
Underlying kidney disease, *n* (%)	[*n* = 120] *	[*n* = 44] *	[*n* = 76] *	0.851
Hypertensive or diabetic nephropathy	18 (15)	6 (14)	12 (16)	
Glomerulonephritis or other glomerular disease	40 (33)	15 (34)	25 (33)	
Polycystic kidney disease or other tubulointerstitial disease	24 (20)	8 (18)	16 (21)	
Congenital anomalies	18 (15)	6 (14)	12 (16)	
Other or unknown cause	20 (17)	10 (20)	11 (14)	
Hypertension (>140/90 mmHg), *n* (%)	[*n* = 119] *		[*n* = 73] *	0.735
Yes	95 (80)	36 (78)	59 (81)	
No	24 (20)	10 (22)	14 (19)	
Dialysis, *n* (%)	[*n* = 121] *		[*n* = 75] *	0.380
Yes	120 (99)	45 (98)	75 (100)	
No	1 (1)	1 (2)	0 (0)	
Duration of dialysis (months), median (range)	[*n* = 119] *		[*n* = 73] *	**0.017**
	47 (3–212)	36 (7–212)	54 (3–169)	
Donor type, *n* (%)				0.649
Living donor	5 (4)	1 (2)	4 (5)	
Deceased donor	118 (96)	45 (98)	73 (95)	
Calcineurin inhibitor, *n* (%)	[*n* = 110] *	[*n* = 42] *	[*n* = 68] *	**<0.001**
Tacrolimus	51 (46)	31 (74)	20 (29)	
Cyclosporine	59 (54)	11 (26)	48 (71)	
Everolimus, *n* (%)	[*n* = 68] *	[*n* = 21] *	[*n* = 47] *	0.423
Yes	7 (10)	1 (5)	6 (13)	
No	61 (90)	20 (95)	41 (87)	
Glucocorticoids, *n* (%)	[*n* = 115] *	[*n* = 43] *	[*n* = 72] *	>0.999
Yes	115 (100)	43 (100)	72 (100)	
No	0 (0)	0 (0)	0 (0)	
Antimetabolite, *n* (%)	[*n* = 115] *	[*n* = 43] *	[*n* = 72] *	0.669
Yes	109 (95)	40 (93)	69 (96)	
No	6 (5)	3 (7)	3 (4)	

Significant *p* values (<0.05) in bold. * = Variables with missing values. NA = not applicable.

**Table 2:** Characteristics of tremor.

	All (*n* = 46)	Female (*n* = 26)	Male (*n* = 20)	*p* Value
Tremor type, *n* (%)	[*n* = 45] *	[*n* = 25] *		0.859
Action	22 (49)	12 (48)	10 (50)	
Rest	10 (22)	5 (20)	5 (25)	
Action and rest	13 (29)	8 (32)	5 (25)	
Tremor distribution, *n* (%)	[*n* = 45] *	[*n* = 25] *		0.199
Focal/segmental	36 (80)	18 (72)	18 (90)	
Generalized	9 (20)	7 (28)	2 (10)	
Time of onset (days), *n* (%)	[*n* = 44] *	[*n* = 25] *	[*n* = 19] *	0.714
1–7	29 (66)	15 (60)	14 (74)	
8–30	11 (25)	7 (28)	4 (21)	
31–180	4 (9)	3 (12)	1 (5)	
Duration of symptoms, *n* (%)	[*n* = 44] *	[*n* = 24] *		0.659
<1 month	7 (16)	5 (21)	2 (10)	
1–6 months	5 (11)	3 (12)	2 (10)	
6–12 months	31 (71)	15 (63)	16 (80)	
>12 months	1 (2)	1 (4)	0 (0)	
Activities of daily living, *n* (%)	[*n* = 45] *	[*n* = 25] *		0.059 **
Normal	25 (55)	12 (48)	13 (65)	
Mild impairment	12 (27)	6 (24)	6 (30)	
Moderate impairment	5 (11)	4 (16)	1 (5)	
Severe impairment	3 (7)	3 (12)	0 (0)	

* Variables with missing values. ** Analyzed for “No impairment” or “Mild impairment” versus “Moderate impairment” or “Severe impairment”.

## Data Availability

The original contributions presented in this study are included in the article/Supplementary Materials. Further inquiries can be directed to the corresponding author.

## Supplementary Materials

The following supporting information can be downloaded at: https://www.mdpi.com/article/10.3390/neurolint18030056/s1, Figure S1: Distribution of sex and calcineurin inhibitor in the “No tremor” versus the “Tremor” group; Figure S2: Characteristics of tremor with respect to age at transplant; Table S1: Multiple logistic regression analysis of potential predictors of post-transplant tremor; Table S2: Characteristics of tremor with respect to the choice of calcineurin inhibitor.

## Abbreviations

The following abbreviations are used in this manuscript:

QoL	Quality of life
CNI	Calcineurin inhibitor
