# Supplementary material for: Post-Transplant Tremor: Characteristics and Differences Based on Sex and Post-Transplant Therapy
Source: Neurol Int. 2026 Mar 17;18(3):56. doi: 10.3390/neurolint18030056 (PMC13029305; doi:10.3390/neurolint18030056)
Supplement: Supplementary file 1 [file neurolint-18-00056-s001.zip › Telarovic_PosttransplantTremor_SupplementaryTableS1.pdf]

Supplementary Table S1. Multiple logistic regression analysis of potential predictors of posttransplant tremor.

	OR (95% CI)	*p* value	VIF
**Age at transplantation**	0.961 (0.924-0.998)	**0.041**	1.091
**Female sex**	2.607 (1.424-10.190)	**0.009**	1.047
**Duration of dialysis**	0.991 (0.977-1.004)	0.175	1.161
**Use of tacrolimus**	3.782 (2.554-18.130)	**<0.001**	1.102

Significant *p* value (< 0.05) in bold. OR = odds ratio. CI = confidence interval. VIF = variance inflation factor.
