# Supplementary material for: Post-Transplant Tremor: Characteristics and Differences Based on Sex and Post-Transplant Therapy
Source: Neurol Int. 2026 Mar 17;18(3):56. doi: 10.3390/neurolint18030056 (PMC13029305; doi:10.3390/neurolint18030056)
Supplement: Supplementary file 1 [file neurolint-18-00056-s001.zip › Telarovic_PosttransplantTremor_SupplementaryTableS2.pdf]

Supplementary Table S2. Characteristics of tremor with respect to the choice of the calcineurin inhibito

	Cyclosporine (n = 11)	Tacrolimus (n = 31)	*p* value
**Tremor type, n (%)**			0.727
**Action**	6 (55)	15 (48)	
**Rest**	3 (27)	6 (19)	
**Action & rest**	2 (18)	10 (33)	
**Tremor distribution, n (%)**	[n = 9]*	[n = 30]*	0.654
**Focal/segmental**	8 (89)	22 (73)	
**Generalized**	1 (11)	8 (27)	
**Time of onset (days), n (%)**	[n = 10]*	[n = 28]*	0.537
**1-7**	6 (60)	20 (72)	
**8-30**	2 (20)	6 (21)	
**31-180**	2 (20)	2 (7)	
**Duration of symptoms, n (%)**		[n = 30]*	0.101
**<1 month**	0 (0)	6 (20)	
**1-6 months**	0 (0)	5 (17)	
**6-12 months**	11 (100)	18 (60)	
**>12 months**	0 (0)	1 (3)	
**Activities of daily living, n (%)**			0.654**
**Normal**	7 (64)	16 (51)	
**Mild impairment**	3 (27)	9 (29)	
**Moderate impairment**	1 (9)	3 (10)	
**Severe impairment**	0 (0)	3 (10)	

Significant *p* value (< 0.05) in bold. * Variables with missing values. ** Analyzed for “No impairment” or “Mild impairment” versus “Moderate impairment” or “Severe impairment”.
