# Supplementary figures and images for: Post-Transplant Tremor: Characteristics and Differences Based on Sex and Post-Transplant Therapy
Source: Neurol Int. 2026 Mar 17;18(3):56. doi: 10.3390/neurolint18030056 (PMC13029305; doi:10.3390/neurolint18030056)

**A****Sex**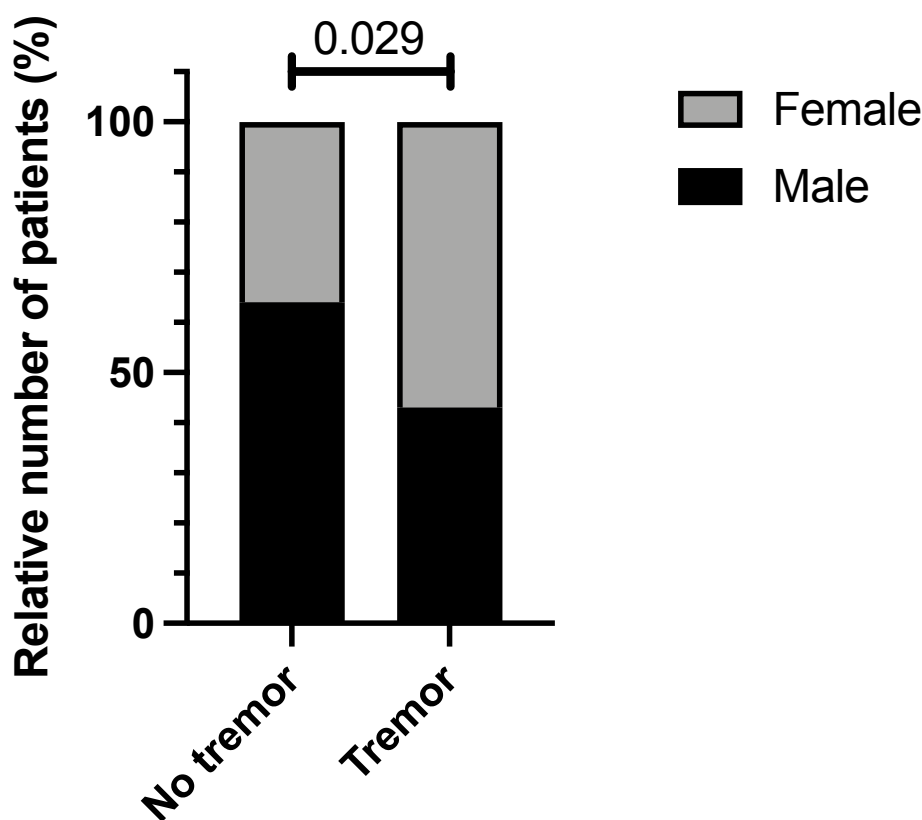**B****Calcineurin inhibitor**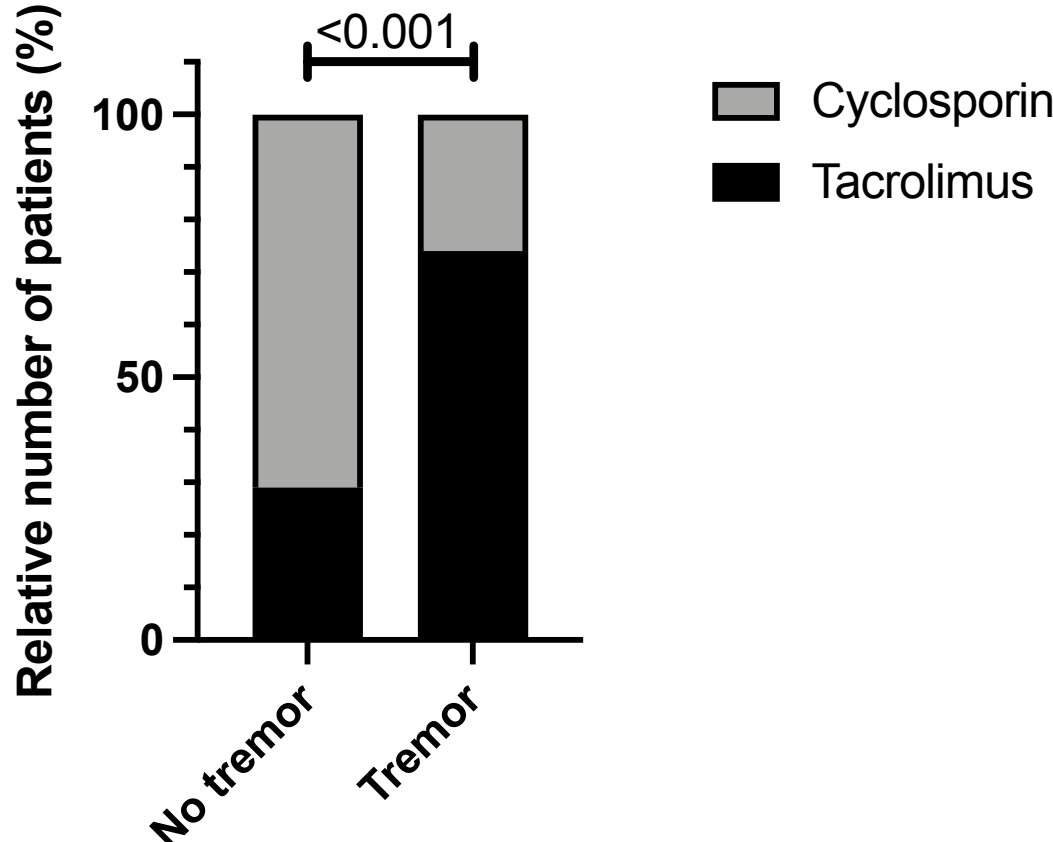

Supplement: Supplementary file 1 [file neurolint-18-00056-s001.zip › Telarovic_PosttransplantTremor_FigureS1.pdf]

**A**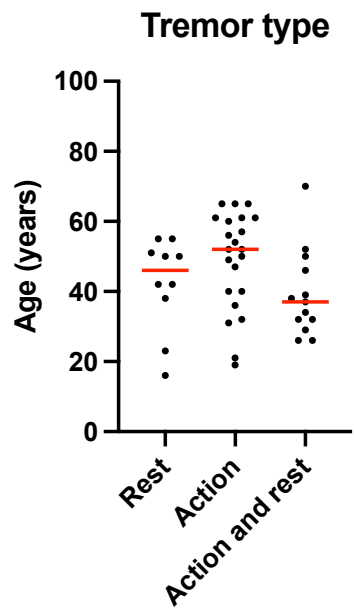**B**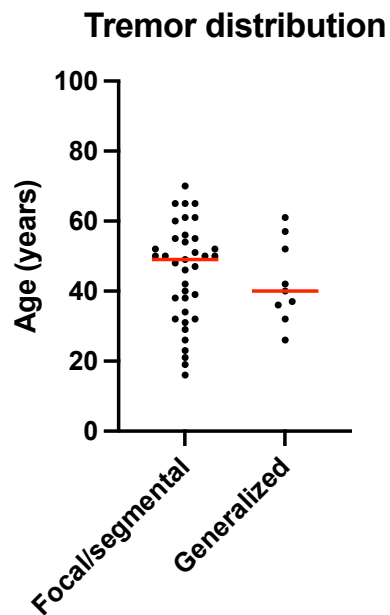**C**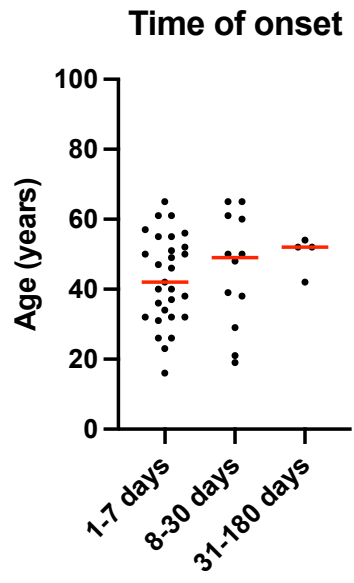**D**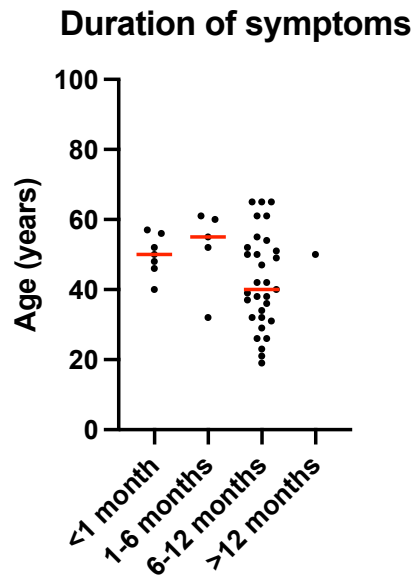**E**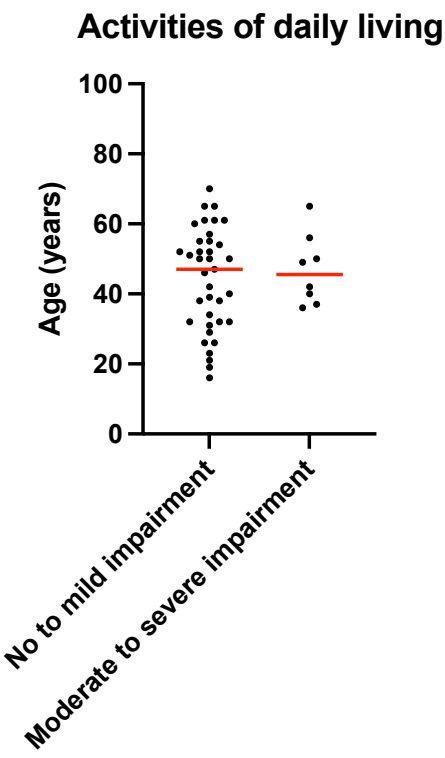

Supplement: Supplementary file 1 [file neurolint-18-00056-s001.zip › Telarovic_PosttransplantTremor_FigureS2.pdf]
